# Fabrication and improved response of ZnO-CdO composite films under different laser irradiation dose

**DOI:** 10.1038/s41598-022-13767-0

**Published:** 2022-06-16

**Authors:** Rayees Ahmad Zargar

**Affiliations:** Department of Physics, Baba Ghulam Shah Badshah University, Rajouri, J&K 185234 India

**Keywords:** Materials science, Physics

## Abstract

Promising Zinc Oxide (ZnO) and Cadmium Oxide (CdO) alloy (ZnO-CdO) films were fabricated on glass substrates by screen printing route for optoelectronic applications. The Nd:YAG green diode laser of wavelength 532 nm and laser fluence of 1.8 J/cm^2^ was used to irradiate the fabricated films at room temperature. The characterization of these films were systematically studied by means of X-ray diffraction (XRD), UV–vis, photoluminescence (PL), Raman spectroscopy and two probe method for conductivity measurement. The XRD pattern shows that all the films were well crystallized with maximum diffraction of (101) plane and mixed phases of ZnO and CdO were detected. The structure, space group and other crystal related parameters were confirmed from Rietveld refinement of XRD data. The basic optical parameters (band gap, refractive index and extinction coefficient) have been estimated using absorbance spectra. The PL spectrum of ZnO-CdO composite films exhibits red shift and blue- green emissions shift upon laser irradiation were confirmed from CIE 1931 diagram. The Raman spectroscopy indicates that the quality of the ZnO-CdO films was increased while their structure defects were increased. DC conductivity measurement confirms semiconductor behaviour. All the parameters such as particle size, optical constants, colour emission and activation energy have been significantly improved upon laser irradiations dose of 1.8 J/cm^2^ for different durations of time. This study could be appropriate for optoelectronic applications.

## Introduction

Recently, intense research on low cost fabrication and laser irradiation has emerged as an efficient method for modifying the structural, optical and electrical properties of the material. Compared with the classic furnace annealing method, the laser irradiation technique has distinct advantages, including fast crystallization at room temperature, low melting point substrates, reduced thermal exposure of the sample and increasing charge carriers^1–3^. The interaction of a laser beam and a solid material is generally characterized by one or more of the four well-known processes which can occur simultaneously: reflection, absorption, scattering and transmission. However, only the absorbed photons in a laser–matter interaction can alter the physical as well as chemical properties of any material^4^. Ternary metal oxide (TMO) alloys have shown significant interest for R&D community mainly due to their usage in the field of energy storage, chemical sensing, and photonic devices^5–7^.

In this direction ZnO and its alloy are most preferable for the fabrication of optoelectronics devices due to its special characteristics like huge exciton binding energy(60 meV) wide direct band gap (3.37 eV) and better thermal stability, low cost, strong radiation hardness, etc^8,9^. The key feature of optoelectronics is to vary, optimize, and realize desired value of band gap energy of ZnO by alloying with Cadmium oxide (CdO)^10^. Since CdO has lower band gap (2.38 eV) than that of ZnO, and higher Cd doping generally reduce the band gap and emit light in the visible range of optical spectra^11^. Further, it is interesting to observe the degree of changes in the different properties of ZnO-CdO composite coated films upon laser irradiation. Various researchers have prepared ZnO-CdO thin films by different techniques such as molecular beam epitaxy^12^, electro-deposition^13^, sol-gel^14^, spray pyrolysis^15^ and screen printing^16^.

In comparison to other techniques, Screen printing is considered as one of the scalable printing techniques and is used to deposit materials onto large-area substrates with requires less time for fabrication^11^. The advantage of its simplicity, scalability and environment-friendly process, shows tremendous potential for fabrication of electronic devices at very low cost. To the best of my knowledge screen printed laser irradiated ZnO-CdO films have not been reported so for. In this paper the author has reported first time the effect of laser irradiation on screen printed ZnO-CdO coated films. The irradiation effects of the laser on the structure, optical and electrical properties of the films were investigated. Based on the experimental results, the presenting paper appeals that post laser irradiation treatment effectively modifying the properties of ZnO-CdO coated films and satisfy the requirement of low temperature in fabrication of solar cell, LCD and so on.

In this paper, the detailed investigation on the effect of sintering on structural, optical and electrical characteristic properties of screen printed ZnO-CdO thick films is carried out. These studies suggest that laser irradiated ZnO-CdO thick films have suitable band gap with minimal number of defects with least strain. Cadmium (x = 0.20) stoichiometric concentration is used for for narrowing ZnO band gap and it red shifts to green region with longer wavelength^16,17^. These studies will help in designing cost effective optoelectronic devices.

## Experimental detail of (ZnO)_0.80_(CdO)_0.20_ coated films:

Fine powders of pure Zinic oxide(ZnO), Cadmium oxide(CdO) and Zinc chloride (ZnCl_2_) were purchased from Merck and Sigma–Aldrich company with purity of 99.99%. The ethylene glycol (C_2_H_6_O_2_) is used here as binder and the calculation for composition is taken as per previous study^16^. The final homogeneous prepared paste was used for the preparation of three samples via screen printing on pre cleaned glass substrates. Before film fabrication, the glass substrates were cleaned with distilled water followed by acetone. The screen printed films were kept on hot plate at 100 °C for 1 h in order to overcome the cracking of the samples. Further the samples were annealed at about 500 °C for 10-min in a temperature controlled furnace that has been reported earlier by the authors^17^. Out of three samples two were irradiated by using Nd: YAG laser of wavelength 532 nm with a 10 Hz repetition rate. The incident laser energy was set to be 354 mJ.

The laser fluence was calculated by using the following formula,

1 $${\text{Fluence }} = \frac{E}{A}$$

Here, E (mJ) is energy of incident laser beam and A (cm^2^) is area or the beam spot size for single pulse irradiation and was about 0.1963 cm^2^ in present case. Therefore, the fluence for different durations of time is 1.8 J/cm^2^.

**Figure 1:**
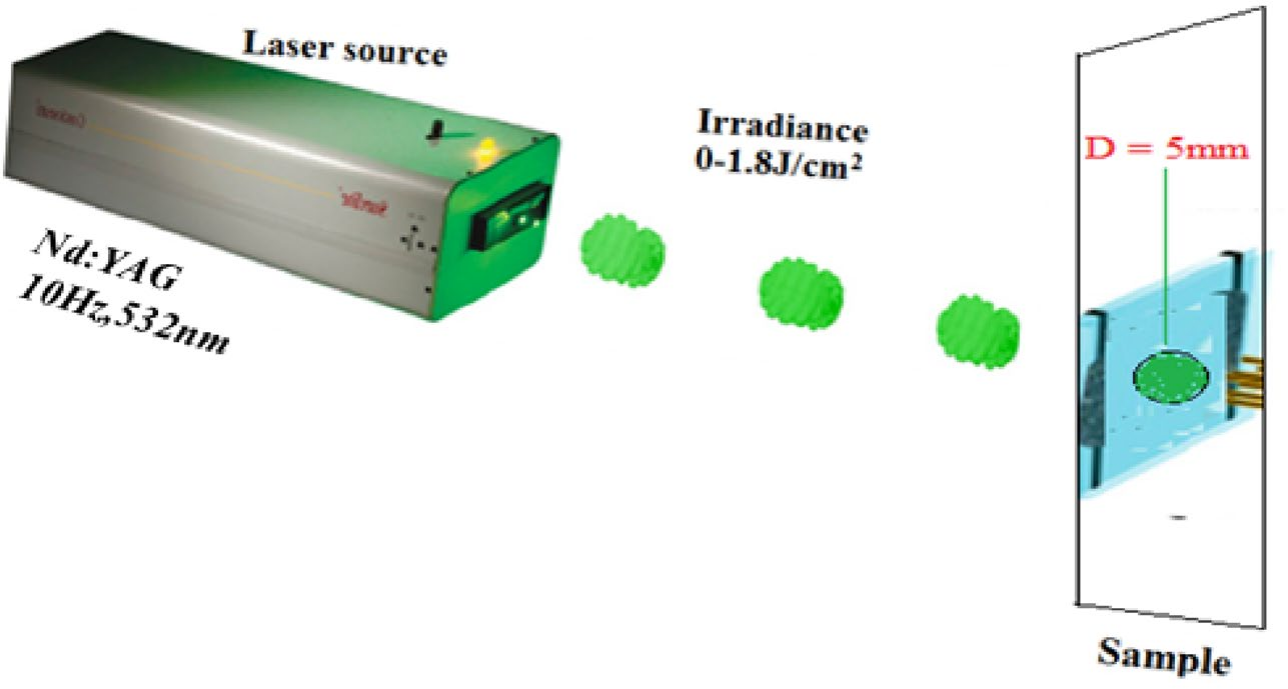
Nd :YAG Green laser set-up diagram used for irradiating the ZnO-CdO coated films.

The schematic set-up diagram used for irradiating the ZnO-CdO coated films is depicted in Fig. 1.

## Characterization techniques used

For microstructural study a Rigaku Miniflex/200 X-ray diffractometer with Cu-Kα (λ = 1*.*5405 Å) radiation with Rietveld full proof programming was used to investigate the phase and crystallinity of the samples and were scanned between 30° to 70° angular range. The optical absorbance and reflectance spectra’s were measured in 300–900 nm range for optical analysis using Hitachi-3400 5300spectrometer with resolution 1 nm. Raman spectra were excited with the 532 nm line of a laser at an incident power of 10mW Ar laser (Lab Ram HR-800 System) with resolution 3.5 cm^−1^ and obtained in the range 100–1500 cm^−1^ was used to investigate the structure and quality of produced films. Photoluminescence emission of all films was measured using a He–Cd laser with an excitation wavelength of 325 nm (Kimmon, Japan). The sample thickness was measured by done Taylor Hobson(Taylor step UK) instrument and thickness of the film is found to be ~ 3 µm. The standard two probe set up (Keithley electrometer-6517A) was used for measuring the I-V and DC resistivity of samples.

## Results and discussions

### Crystal structure and phase analysis

**Figure 2:**
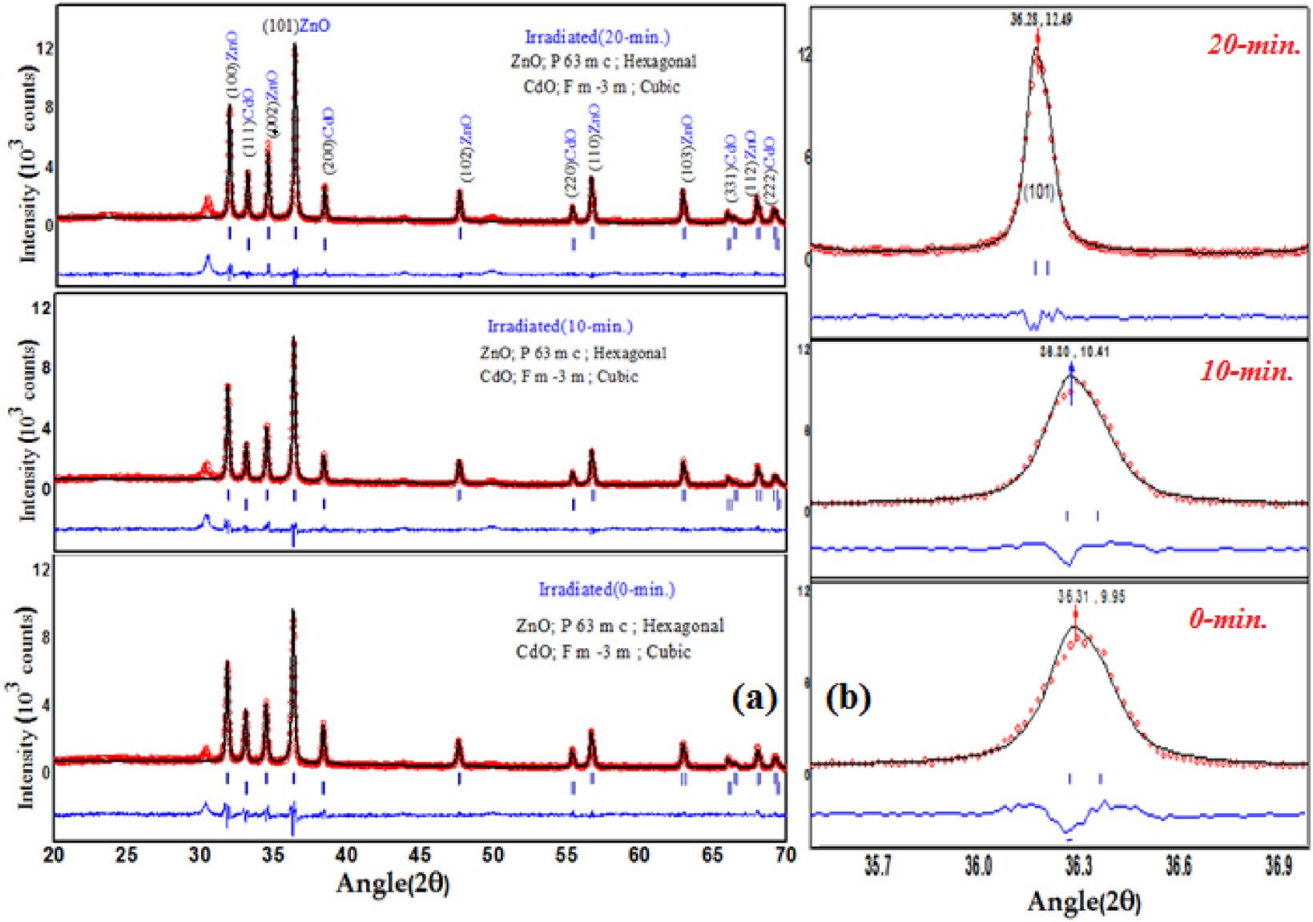
(**a**) Rietveld fiiting of XRD patterns of as-grown and irradiated ZnO-CdO coated films. (**b**) Enlarged area of 2θ from 35° to 37° peak corresponding to (101) plane showed shift under (10-min and 20-min) irradiation dose.

The discussed structural properties of the as-grown and irradiated ZnCdO screen printed films were examined using X-ray diffraction (XRD) technique. The phase-segregation into crystalline hexagonal mixed metal ZnCdO(*P*63*mc*) and cubic CdO(*Fm*3.*m*) phases was detectable when investigated with Rietveld refinement technique and is depicted in Fig. 2a. The diffraction pattern consists of peaks(100), (002), (101), (102), (110), (103) and (112) for ZnO hexagonal wurtzite where as peaks (111), (200), (220), (331) and (222) represents CdO cubic respectively. From XRD pattern (101) is the maximum diffraction of plane occur among them. All the peak positions have been verified as per JCPDS card nos. (36–1451:ZnO) and (05–0640:CdO) respectively except one peak at 30° which may be attributed due to the presence of ZnCl_2_ as ZnCl_2_ is used as adhesive in ZnO-CdO films . Thus, XRD spectra consist of only ZnO and CdO diffraction peaks and don’t have any metallic Zn or Cd peaks, it means that Zn and Cd are completely oxidized^18^.

The mean crystallite size (D_p_) was calculated according to Debye–Scherrer formula^18:^

2 $$D_{p} = \frac{0.94\lambda }{{\beta {\text{Cos}} \theta }}$$

where D_p_ is the diameter of the particles, λ = 1.542 Å is the wavelength of X-ray, β represents full-width at half-maximum (FWHM) of the main diffraction peak and θ is half of the diffracted angle (in radians). The average crystallite size was found to be about 34.23–39.33 nm of the samples and are mentioned in Table 1.

**Table 1:** XRD refinement parameters and structural information of the ZnO-CdO coated films.

Parameters	Irradiated (0-min)	Irradiated (10-min)	Irradiated (20-min)
Structure or phase fraction and Space group	ZnO; P 63 m c; 86% Hexagonal CdO; F m -3 m; 14% Cubic	ZnO; P 63 m c; 88% Hexagonal CdO; F m -3 m; 12% Cubic	ZnO; P 63 m c; 90% Hexagonal CdO; F m -3 m; 10% Cubic
Mean crystallite size(D_p_)	34.23 nm	35.53 nm	39.33 nm
Lattice parameter	a = b = 3.252 Å (± 0.001) and c = 5.242 Å(± 0.017) for (ZnO) & a = 4.696 Å(± 0.05) for (CdO)	a = b = 3.241 Å (± 0.001) and c = 5.238 Å(± 0.017) for (ZnO & a = 4.591 Å(± 0.05) for (CdO)	a = b = 3.233 Å (± 0.001) and c = 5.220 Å(± 0.017) for (ZnO) & a = 4.587 Å(± 0.05) for (CdO).
Fitting factors	χ^2^** = **1.83 (ZnO) χ^2^** = **1.78 (CdO)	**χ**^**2**^** = **1.87 (ZnO) **χ**^**2**^** = **1.81 (CdO)	**χ**^**2**^** = **1.93 (ZnO) **χ**^**2**^** = **1.87 (CdO)

Further confirmation of the structure, phase, space group, lattice parameters and goodness of fit index was performed using Foolproof Software are also mentioned in Table 1. It is observed that the characteristic peak (101) moved towards lower angle from ${36}{\text{.313}}^{ \circ }$ to ${36}{\text{.281}}^{ \circ }$ while the Full width at Half Maxima (FWHM) decreased from ${0}.47^{ \circ }$ to ${0}.361^{ \circ }$ with laser irradiation as demonstrated in Fig. 2b. It is an indication of increasing particle size and decreasing of lattice parameters of the mixed structure. However the intensity for CdO peak at (111) and (200) plane for 10-min is less than that of 0-min and again it is increased for 20-min irradiation has been observed from XRD spectra. This may caused by the oxidation of dopant in ZnO-CdO film during the laser interaction for the reason of thermal and photochemical effects of Nd-YAG laser, whereas at higher laser irradiation provide sufficient thermal energy to adjust the order of crystal lattice and hence intensity start increasing. The increase of crystal size as well as slight variation of lattice parameters was observed, which indicates the existence of compressive stress in the irradiated films due to the high creation of defects. The fitting quality of experimental data have been assessed by computing the goodness of fit parameter ($\chi^{2} < 2$), this lower value of $\chi^{2}$ justifies the goodness of refinement^19^. These results lead to the fact that composite grains have been decomposed because the formation of particulates is target material-dependent, and when the target material contains oxygen gas, the irradiated surfaces might become oxygen- free by laser heating. This could change the fractions of the hexagonal and cubic material components as a result this could change the lattice parameters for both phases^20^.

### UV–visible spectroscopy

**Figure 3:**
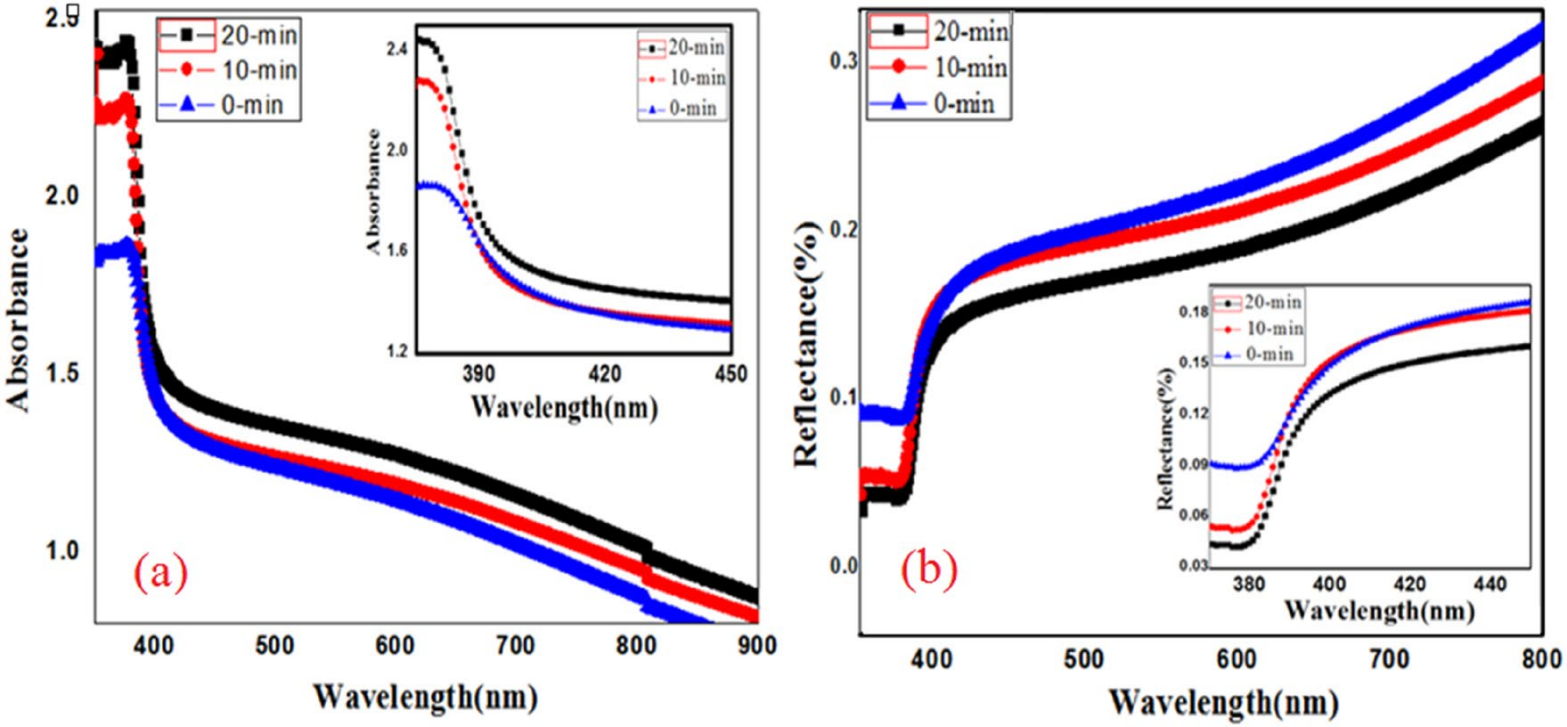
(**a**) Absorbance, (**b**) Reflectance spectra’s along with their fitted insets for clearer of red shift of ZnO-CdO coated films under (10-min and 20-min) irradiation dose.

UV–visible spectroscopy is a fundamental characterization technique to study the desired optical information of the material regarding electronic transition (wavelength and wave number) and its intensity. The current study express a systematic calculation of optical parameters such as band gap, refractive index, and extinction coefficient with help of standard relations and here absorbance and reflectance spectra’s of the material are taken by using UV–visible spectrophotometer (Hitachi-3400) in the wavelength range 350–900 nm, Fig. 3a,b the films exhibits higher absorbance and lower reflectance between 350–450 nm. This variation is obvious, because the absorbance and reflectance is the opposite phenomena. The average absorbance increases upon laser irradiation (10-min and 20-min) where as reflectance decreases in the visible region and both the spectra’s shows red shift and is clearly shown in the inset of Fig. 3a,b. The decrease of reflectance may be due to the existence of localized energy levels between the valence and conduction band^21^.

For the direct transition in a semiconductor, the variation of the absorption coefficient α is determined according to the following relation^22^.

3 $$\alpha hv \propto (hv - E_{g} )^{1/2}$$

**Figure 4:**
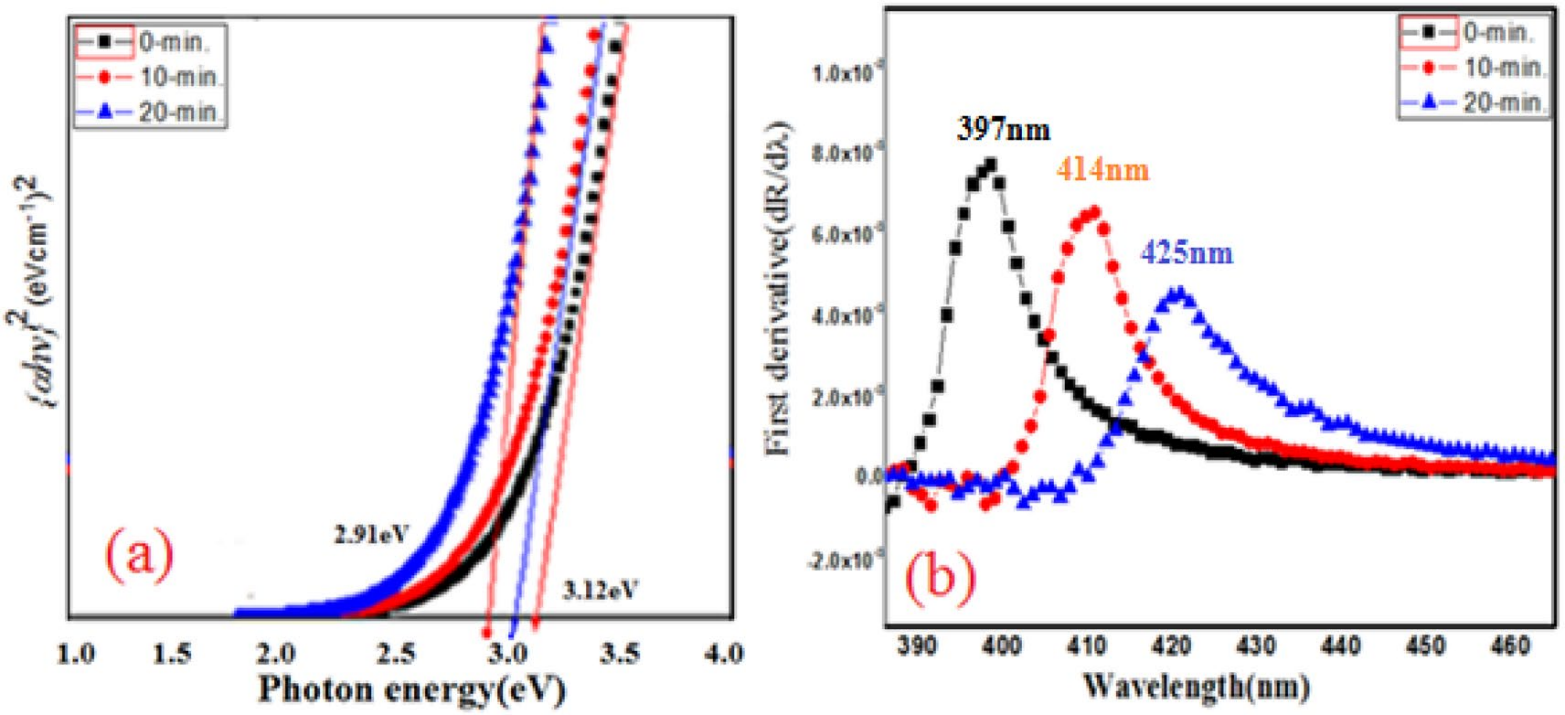
(**a**) Tauc plot for energy bad gap determination and (**b**) First derivative plots of Reflectance of as-grown and irradiated ZnO-CdO coated films.

Here E_g_ = optical band gap, α = absorption coefficient and h*v* = incident photon energy. Figure 4a shows the variation plot of ($\alpha h\nu$)^2^ vs photon energy ($h\nu$) which is a straight line that corresponds to direct transition and is called Tauc’s plot which gives optical band gap (E_g_) of samples. The optical band gaps of as grown film and irradiated films (10-min, and 20-min) are estimated to be about 3.12, 2.97, and 2.91 eV, respectively. The lower band gap value of ZnO-CdO is 3.01 eV,it is because band gap of CdO is lesser than ZnO, while alloying of Cd, the 5 s electron state of Cd, below the conduction band will become more predominant than the high energy4s state of Zn. This shifts the conduction band edge downwards and result in decrease in band gap^23^.

The estimated absorption band edge of the films was computed from the first derivative of optical reflectance as shown in Fig. 4b. The curves between dR*/*d*λ* and wavelength give a peak that corresponding to the absorption band edge. As seen in Fig. 4b, the peak position of the curves shifts to longer wavelengths, suggesting decreasing of band gap upon laser irradiation. The peak positions varied from 397 to 426 nm for the ZnO-CdO film (0-min) and irradiated films (10-min, and 20-min) films respectively. This decrease of 0.21 eV band gap is due to increase in particle. Hence, the absorbance below 465 nm makes ZnO-CdO film a righteous choice of light detectors from UV to blue region.

Further in order to study the complete optical behaviour of prepared samples, it is very important to pay more attention towards refractive index (*n*) and extinction coefficient. These parameters basically give information about the suitability of material for fabrication of various optoelectronic devices. The refractive index (n) and extinction coefficient (k) were calculated by using below relations^24,25^:

4 $$n = \left( {\frac{1 + R}{{1 - R}}} \right) + \sqrt {\frac{4R}{{(1 - R)^{2} }} - K^{2} }$$

5 $$\kappa = \frac{\alpha \lambda }{{4\pi }}$$

where λ = wavelength of the incident photon, α = absorption coefficient and R = reflectance.

**Figure 5:**
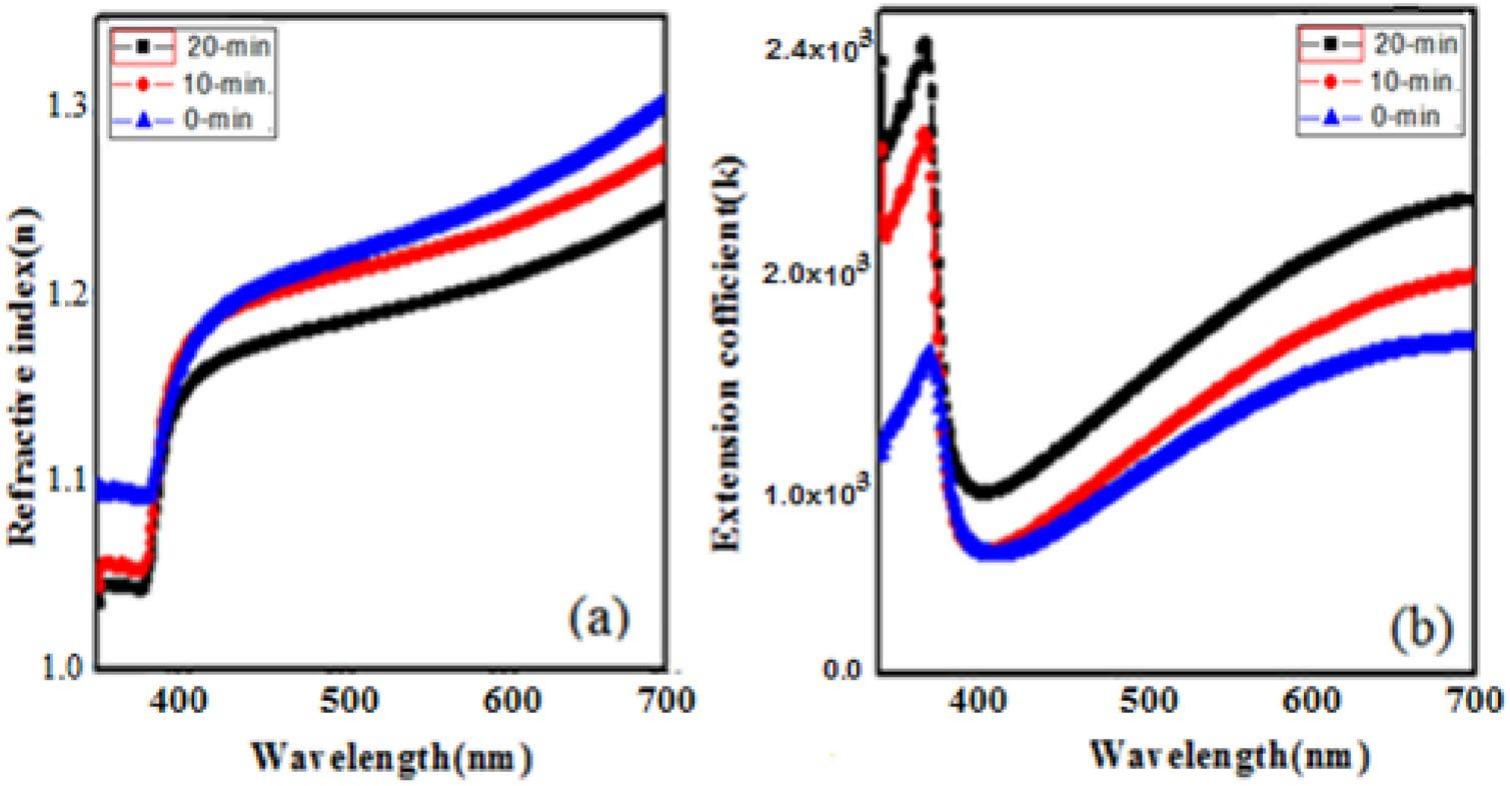
(**a**) Refractive index and (**b**) Extinction coefficient of as-grown and irradiated ZnO-CdO coated films.

Figure 5a shows the variations of refractive indices verse wavelength of ZnCdO coated films. For without irradiated film maximum *n* is 1.3 at 700 nm, while for laser irradiated films, *n* varies from1.27 to 1.21 and shows sharp shift toward higher wavelength. Therefore, it is seen that *n* shows anomalous dispersion below 700 nm, and then followed by normal dispersion after 400 nm. However, the refractive indices overall decreases with increasing irradiation dose due to reduction of reflectance. Whereas Fig. 5b depicts the extinction coefficient of the films. The k represents basically inelastic scattering of the electromagnetic waves in the semiconductor such as the, photoelectric effect, Compton effect and so on^26^. The value of k sharply increases within the band gap regime, remains stable till 400 nm and then shows down fall. Inspection of Fig. 5b shows increase in k upon laser irradiation. Thus, Inspection of Fig. 4b shows similar behaviour of absorbance, which means that the extinction coefficient is absorption coefficient related according to (4).

This variation of k and n within the band gap regime upon photo induced phenomena is consequences of local structural modification that brings the Cd atoms close to the Zn atoms and as a result will be change in the bond length, therefore, surface defects and disorder caused by localized states^27,28^. Hence the lower value of n and higher value of k in the UV–visible region makes them proficient in designing optoelectronic devices.

### Photoluminescence analysis

**Figure 6:**
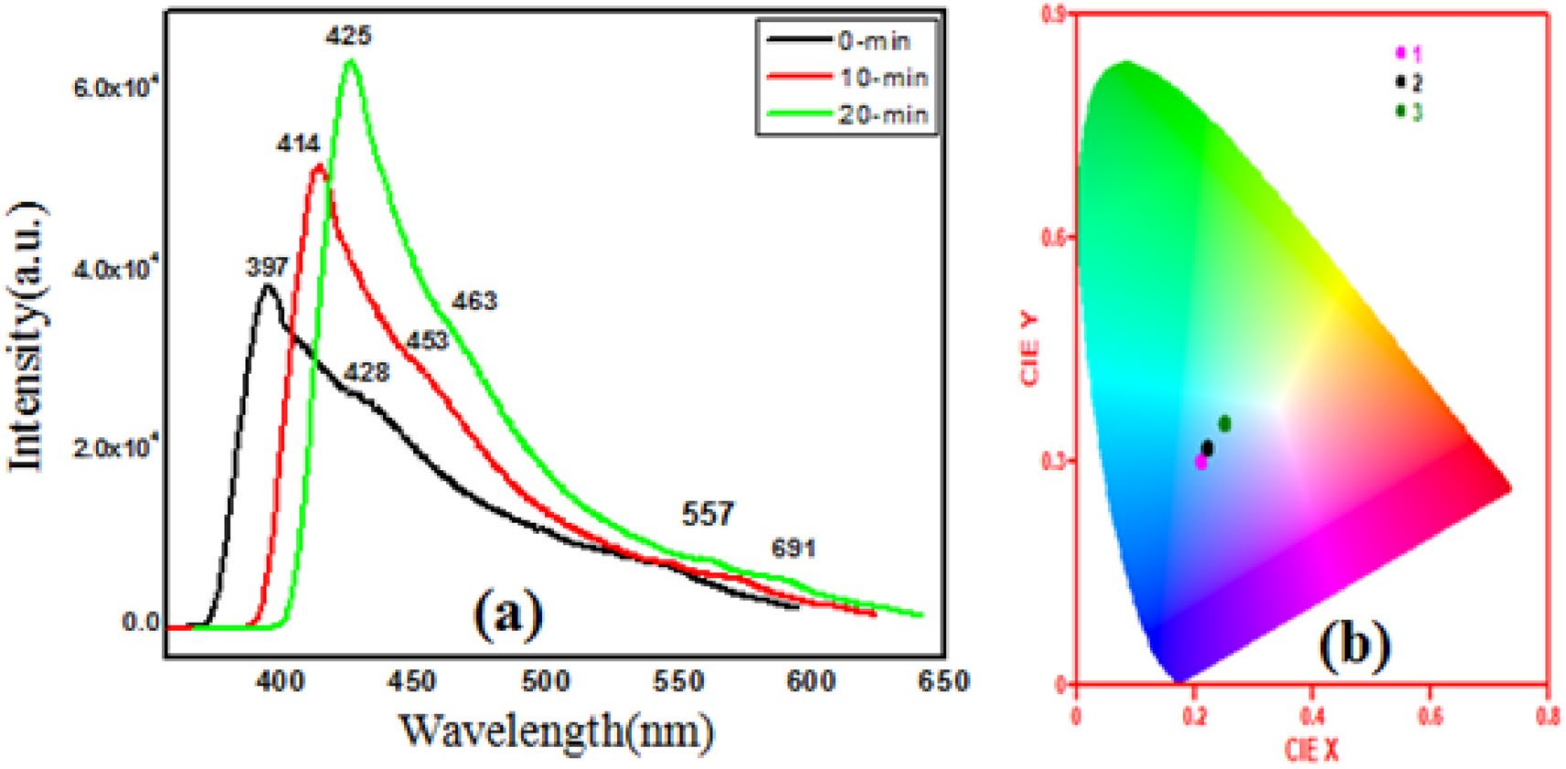
(**a**) PL spectra of of as-grown and irradiated ZnO-CdO coated films. (**b**) CIE diagram for colour identification of ZnO-CdO coated films at (0-min, 10-min and 20-min) respectively.

Photoluminescence study is one of the essential characterizations for recombination and generation of electrons and holes in semiconductors in addition to relative energy position of the sub-band gap and the density of defects^16^. Figure 6a, shows the clear band-edge luminescence spectra with enlarged for as grown and irradiated ZnCdO coated films under the excitation of 325 nm. The ZnO emission is usually classified into two categories: one is the ultraviolet emission near band edge(NBE) in the UV region related to the direct band gap transition and/or free-exciton recombination. The broad blue green emission peak in ~ 450–500 nm superimposed with weak three shoulders is observed with maximum at ~ 428 nm (2.89 eV) due to recombination of electron–hole pairs via exciton-exciton collision process, oxygen vacancies, Zn, Cd interstitials and deep level defects in ZnCdO coated film^29^. Figure 6a, it can be seen that after laser irradiation of two samples at (10-min and 20-min) the UV emission peak of ZnCdO films undergoes shifting from 397 (3.12 eV) to 425 nm (2.91 eV) with increasing of FWHM and shifting of blue green emission peaks were also observed. The increasing of intensity of UV emission peak is linked with the improving of crystalline quality of films^30^. The shifting of peak position in the UV emission after laser irradiation might be attributed to the change in the band gap (Fig. 4b).

A light source emitting in visible region is characterizes by chromaticity color coordinates (x and y) which indicate the expected color (as perceived by normal human eye) of its emissions. To calculate these coordinates a color space diagram is constructed from the emission spectrum and is depicted in Fig. 6b, called Commission International de l’Eclairage (CIE). The chromaticity diagram of ZnCdO coated films at various irradiation times calculated using photoluminescence data. It can be seen from the figure that the Pl CIE coordinates (x,y) of (0.210,0.300), (0.220,0.320) and (0.250,0.350) correspond to (0-min,10-min and 20-min). The coordinates were shifted toward the near white light with the increase in laser irradiation. This was because the emission peaks in Fig. 6a were red-shifted. Thus, this indicates that laser irradiation play a major role in tuning the emission colour of the ZnCdO coated films. This blue–green emission tending to white light upon laser irradiation is a good identification of materials for applications in light emitting devices.

### Raman spectroscopy

**Figure 7:**
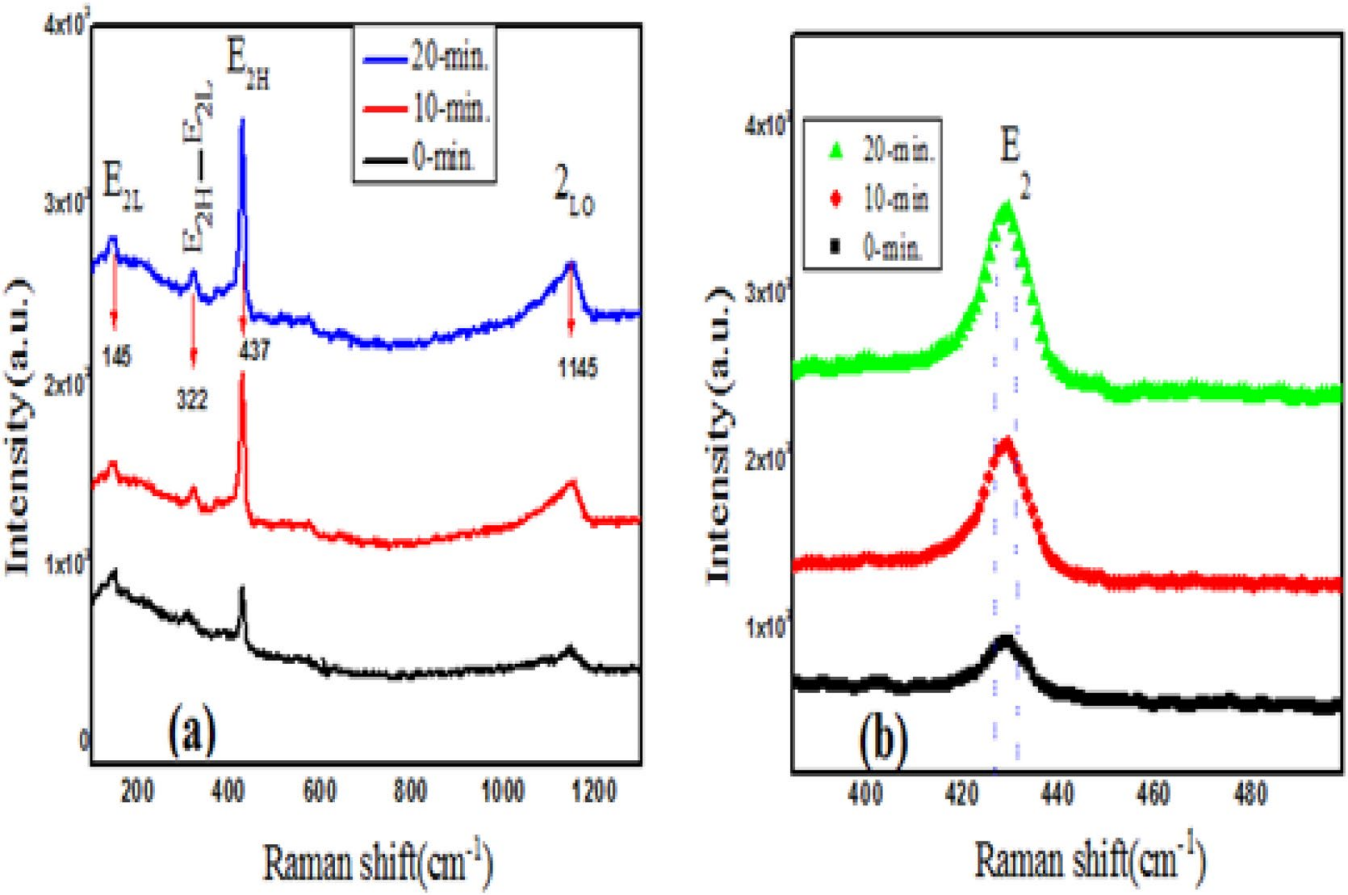
(**a**) Raman spectra of as-grown and irradiated ZnO-CdO coated films. (**b**) Shifting of E_2(H)_ phonon mode films at (0-min, 10-min and 20-min) respectively.

The Raman spectroscopy is usually applied to identify the vibrational modes of material and Fig. 7a depicts the change of structural disorder in as-grown and irradiated films. According to group theory the ZnO has wurtzite structure with $C_{6v}^{4}$ space and A_1_, 2B_1_, E_1_, and 2E_2_ are Brillouin zone center for optical modes^31^. The A_1_ and E_1_ are called polar and Raman modes and are mostly infrared active, while as the two E_2_ modes (E_2(L)_ , E_2(H)_) are nonpolar and in which only Raman is active and B_1_ modes are forbidden in optical spectra^19^. From Fig. 7a the two Raman peaks are clearly visible at about 139 and 437 cm^−1^ related to the E_2(L)_ and E_2(H)_ modes and are detected in all films. The weak peak situated at 322 cm^−1^ is related to the second-order E_2(H)_—E_2(L)_ band of ZnO films and 2L_O_ phonon is observed at ~ 1143 cm^−1^ where as B_1_ modes are usually known as silent modes. Among these peaks, the *E*_2(H)_ mode centred at 437 cm^−1^ is the most intense peak and sharper line-width, which indicates that fabricated films are composed of ZnO with a hexagonal wurtzite structure and good crystal quality^32^. The CdO phonon modes were not identified in the ZnCdO spectra and reason for their absence has been explained by Wang et al.^33^. It has been observed that peak position shifts toward lower wave number and the intensity of films increased upon laser irradiation which indicates the occurrence of structural change. The increase of intensity is linked with the formation of defective bonds which is mentioned in the optical part also.

The relationship between particle size and photon position can be expressed on the basis of Heisenberg uncertainty principle and can be followed as:

6 $$\Delta X * \Delta P \ge \frac{\hbar }{2}$$

where $\Delta X$ = particle size, $\Delta P$ = phonon momentum distribution, and $\hbar$ = Planck’s constant. As the particle size increases, the phonon is not confined within the particle and the phonon momentum distribution decreases. This phonon dispersion causes asymmetric broadening and may lead to a shift of the Raman bands^34^. which is in good agreement with the XRD analysis result. The shifting of *E*_2(H)_ mode towards lower wave number with increase in intensity upon laser irradiation and is shown in Fig. 7b, this behaviour is almost similar with the XRD result.

## Electrical properties of the ZnO-CdO films

**Figure 8:**
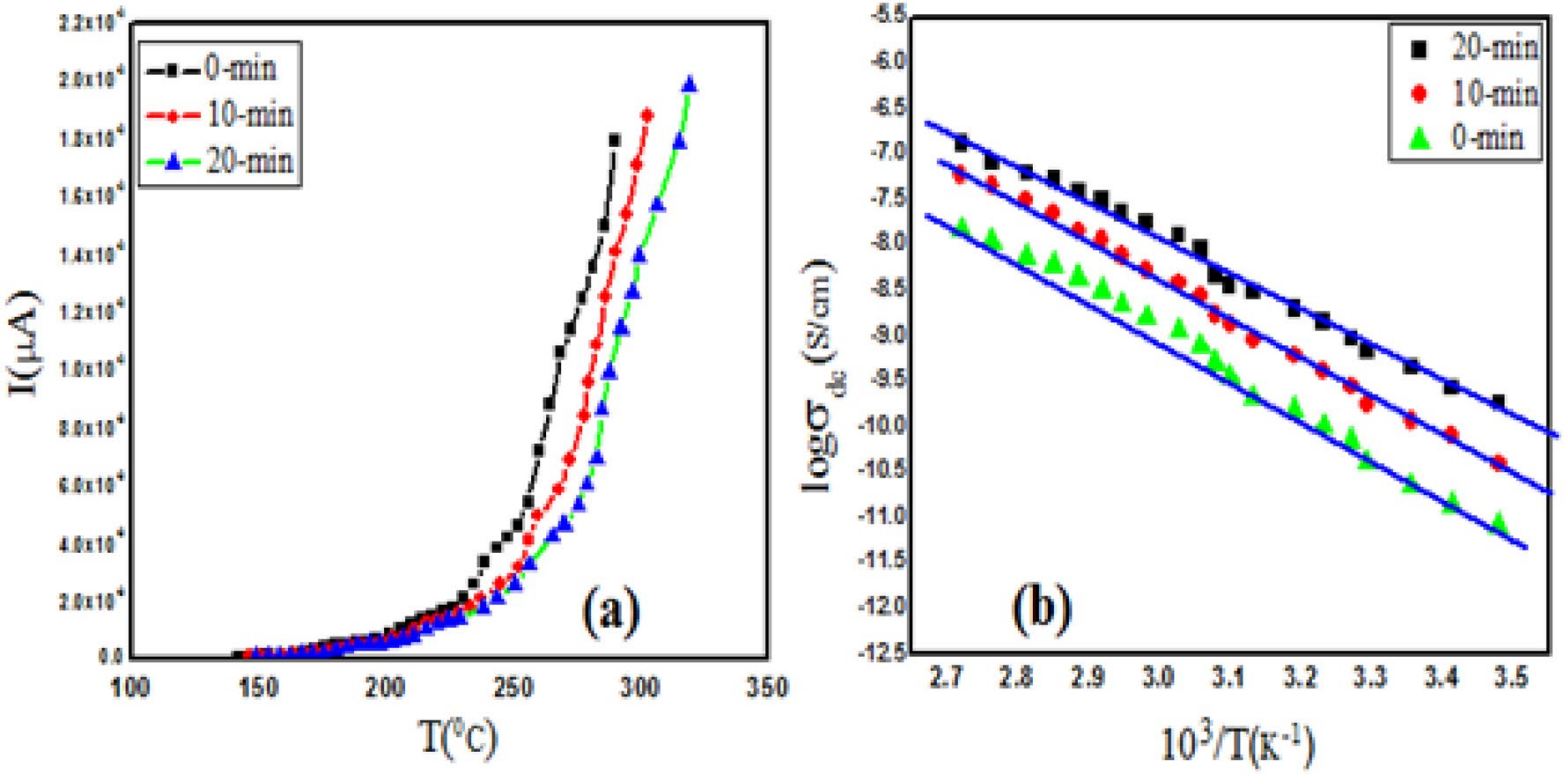
(**a**) Temperature dependence of dark current of as-grown and irradiated ZnO-CdO coated films. (**b**) DC conductivity beahaviour of ZnO-CdO coated films at (0-min, 10-min and 20-min) respectively.

Electrical behaviour of ZnO-CdO coated films play a prominent role for optoelectronic device applications is because of their suitable electrical conductivity due to presence of oxygen vacancies and zinc interstitials in the ZnO lattice^35^. Figure 8a shows the variation of current with temperature for as grown (0-min) and irradiated films (10-min and 20-min) in the temperature range from 100 to 350 °C. The exponentially growth of current(I), raises as the temperature increases as well as irradiation duration which means that thermally activated charge carriers as well as photo induced effect takes place. The current intensity of irradiated films is higher as compared to as grown film and enhances as the laser irradiation increases. It is probably that Nd-YAG laser has not only thermal effect but also the photochemical effect on ZnO-CdO composite films. The phonon energy of the laser can break Zn–O/Cd–O bonds and accumulates more carrier concentrations,thus, the conductivity of the film increases quickly^36^. Figure 8b shows the logarithmic conductivity with inverse temperature plot and is obtained by the help of well-known Arrhenius equation.

7 $$\sigma =\beta exp \left(\frac{-\Delta E}{KT}\right)$$

where $\sigma$ = conductivity, $\beta$ = the pre-exponential factor and E = thermal activation energy k = Boltzmann’s constant (1.38 × 10^−38^ J/K) and T = applied temperature (in Kelvin). It is observed that the films are semiconducting in nature and the conductivity increases with increasing in temperature and the laser irradiation as well. The activation energy represents the location of trap levels below the conduction band and is calculated from the slope of graph. The calculated values of activation energy of grown and irradiated films are 0.31, 0.28, and 0.23 eV, respectively. The decreasing of activation may be hopping of localized levels due to the excitation of carriers from one defect state to another. The conductivity of the as deposited film is $7.9 \times 10^{ - 7}$ S/cm while that of the irradiated films are $7.1 \times 10^{ - 6}$ S/cm and $6.6 \times 10^{ - 5}$ S/cm. The increase in the conductivity of the irradiated films is most likely due to the increase in the concentration of bulk oxygen vacancies that is caused by irradiation^37^.

**Figure 9:**
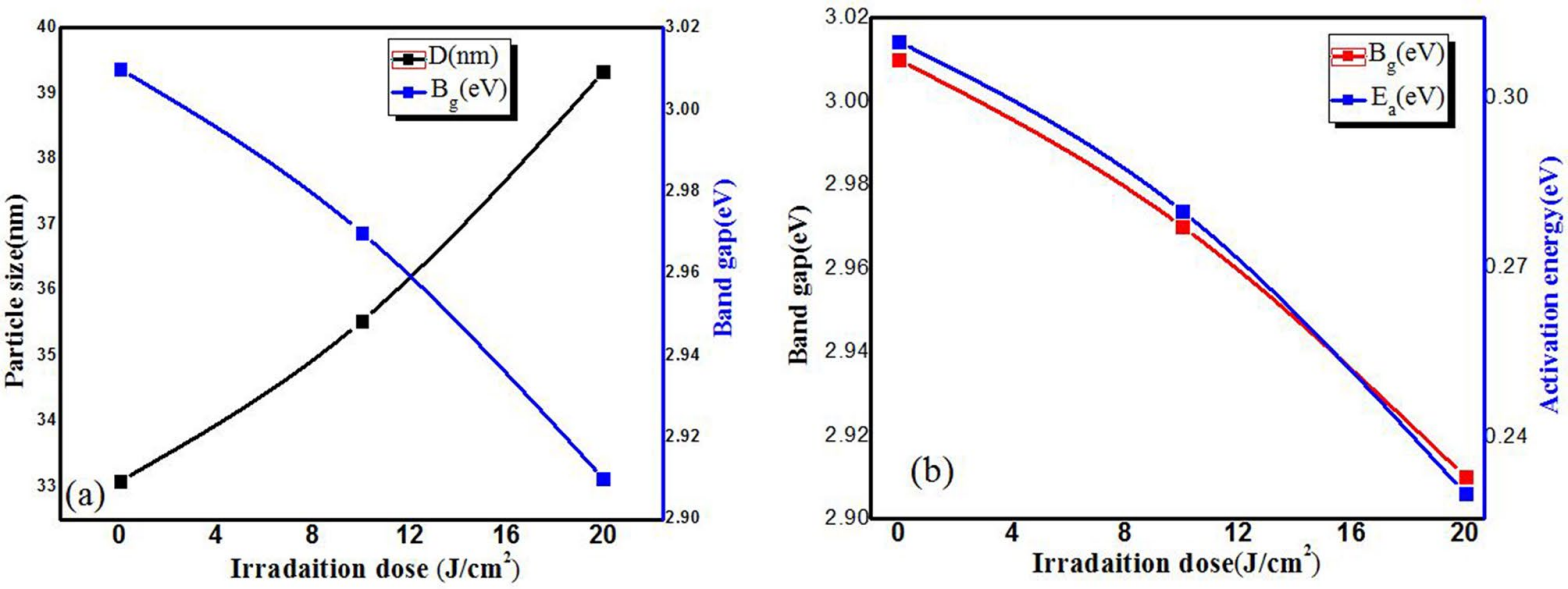
(**a**) Correlation between particle size Vs band gap (**b**) Correlation between band gap Vs activation energy with respect to irradiation dose of ZnO-CdO coated films.

Figure 9a shows better comparison for band gap vs particle with respect to laser irradiation dose in which particle size increases while as band gap decreases, since annealing at a suitable temperature can provide the atoms enough energy to occupy lower energy sites in the crystal lattice, leading to the larger grain size and lower band gap energy. However, both band gap and activation energy decreases with increase in laser irradiation, this means optical and electrical conductivity can be easily controlled simultaneously by changing laser irradiation as shown in Fig. 9b. Therefore, compared with laser irradiation, annealing treatment can improve the structural and opto-electrical qualities of ZnO-CdO thick films.

## Conclusion

Composite coated films of ZnO-CdO paste material have been successfully prepared using simple screen printing technique. The prepared samples have been irradiated by green Nd-YAG laser at 1.8 J/cm^2^ fluence for (10-min and 20-min). The Coexistence of two phases were identified from XRD spectra and found sensitive under the influence of laser light. The direct band gap has found decreased and as well as resistivity, while the carrier concentration and mobility increased, with the increase of under laser energy. The results show that ZnO-CdO coated films have excellent thermal resisting properties. Thus, the laser annealing is an effective way for the modification of materials physical properties and in the future, this technology can be utilized for large-area flexible optoelectronic devices application.
